# Successful treatment of a primary gastric plasmacytoma mimicking intractable gastric ulcer by using high-dose dexamethasone therapy: a case report

**DOI:** 10.1186/s13256-016-0863-1

**Published:** 2016-03-31

**Authors:** Da-yeong Kang, Gee-Bum Kim, Byung-Seok Choi, Jun-won Seo, Hyun-Jong Lim, Ran Hong, Sang-Gon Park

**Affiliations:** 1 Department of Internal Medicine, Chosun University Hospital, 365 Pilmun-daero, Dong-gu, Gwangju, 501-717 Republic of Korea; 2 Department of Medicine, Graduated School of Chosun University, 309 Pilmun-daero, Dong-gu, Gwangju, 501-717 Republic of Korea; 3 Department of Pathology, Chosun University Hospital, 365 Pilmun-daero, Dong-gu, Gwangju, 501-717 Republic of Korea; 4 Department of Internal Medicine, Hemato-oncology, Chosun University Hospital, 365 Pilmun-daero, Dong-gu, Gwangju, 501-717 Republic of Korea

**Keywords:** Plasmacytoma, gastric ulcer, gastric cancer, dexamethasone

## Abstract

**Background:**

Extramedullary plasmacytoma is a plasma cell neoplasm that presents as a solitary lesion in soft tissue. Most extramedullary plasmacytomas involve the nasopharynx or upper respiratory tract. Primary plasmacytoma of the stomach is extremely rare.

**Case presentation:**

A 78-year-old Korean woman presented with epigastric pain for 3 months. She had a history of an intractable gastric ulcer despite repeated endoscopic biopsies and appropriate medical therapy for the ulcer. She underwent another endoscopy and a biopsy was performed for multiple large and deep specimens. Ultimately, primary gastric plasmacytoma was confirmed. However, she and her attendant refused standard local radiotherapy or surgical resection. She came to our emergency room 3 months later with hematemesis due to a large gastric ulcer, despite management with medication for over 3 months at a local clinic. We again recommended local radiation or surgical resection. However, as she was willing to undergo only medical therapy, she was prescribed high-dose dexamethasone. Surprisingly, her ulcer completely regressed and remission was maintained for over 1 year.

**Conclusions:**

We report successful treatment of a rare primary gastric plasmacytoma mimicking intractable ulcer by using high-dose dexamethasone. To the best of our knowledge, this is the first reported case successfully treated with only high-dose dexamethasone.

## Background

Plasma cell neoplasms are commonly divided into four groups: multiple myeloma (MM), plasma cell leukemia, solitary plasmacytoma of the bone (SPB), and extramedullary plasmacytoma (EMP) [1]. Most EMPs involve the upper respiratory tract, including the sinuses, nasal cavity, and nasopharynx; isolated primary plasmacytoma of the stomach is extremely rare [2].

## Case presentation

A 78-year-old Korean woman presented with a complaint of epigastric pain for 3 months. She was diagnosed with atrial fibrillation and ejection fraction-preserved heart failure 5 years earlier. She received regular follow-up observations and took aspirin.

She initially underwent an endoscopy at our clinic and was found to have extensive mucosal erythema with an ulcer crater at the antrum. Pathologic findings revealed chronic gastritis without *Helicobacter pylori* colonization. She underwent gastric ulcer therapy including a proton pump inhibitor (PPI). After 1 month, a follow-up endoscopy showed an unchanged ulcer. A repeat biopsy examination revealed chronic inflammation without *H. pylori* colonization. She was prescribed another PPI with a regimen for *H. pylori* eradication (metronidazole, clarithromycin, PPI). However, her epigastric symptoms continued and she left our hospital.

**Fig. 1:**
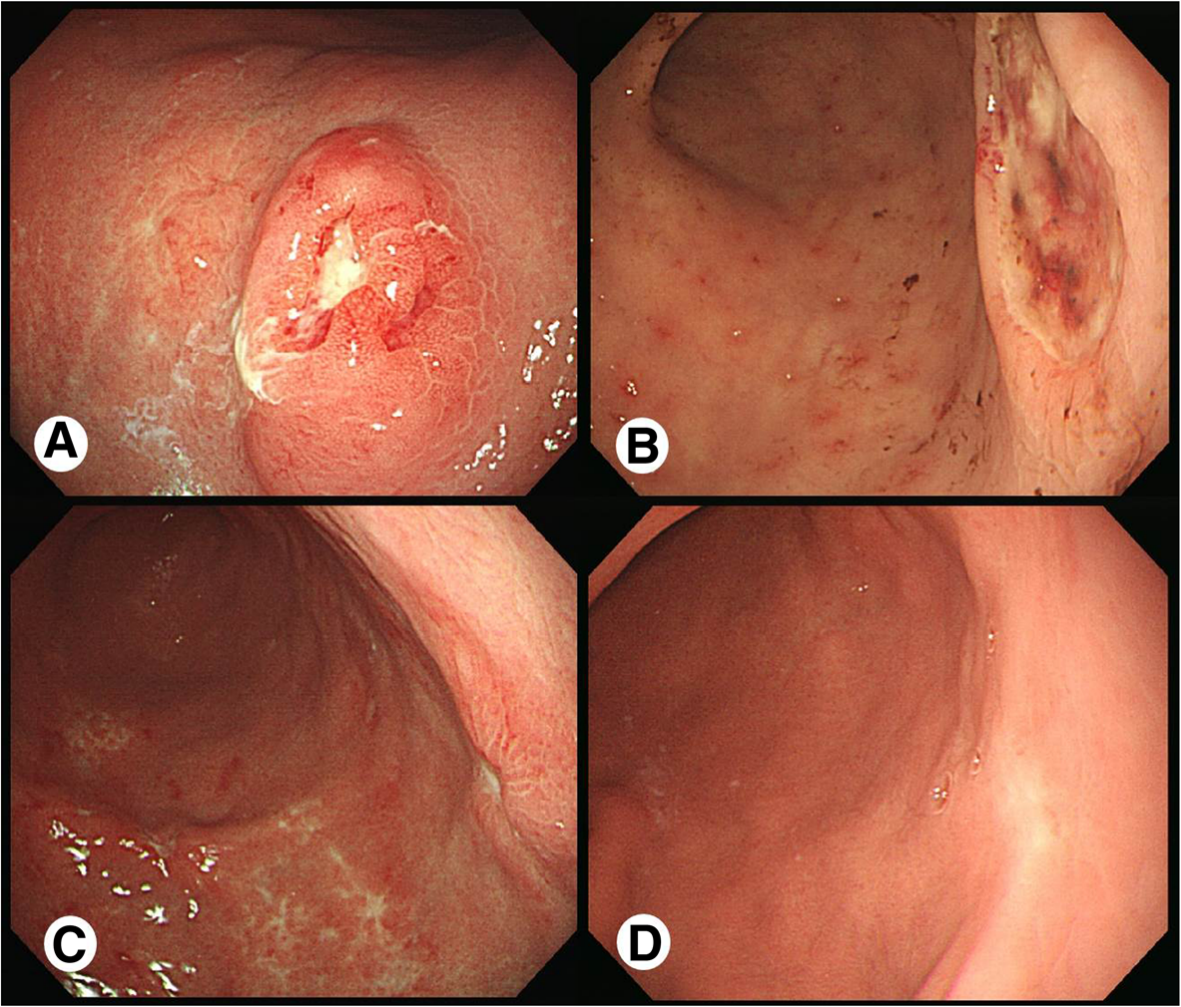
Endoscopic finding. **a** Extensive mucosal erythema with an ulcer crater at the antrum was seen. **b** A large gastric ulcer with a bleeding spot at the antrum was seen 3 months later. **c** After 2 weeks of high-dose dexamethasone, the ulcer lesion showed marked healing. **d** After four cycles of high-dose dexamethasone, a healed ulcer scar replaced the ulcer lesion

A follow-up endoscopy performed 2 months later, despite appropriate and sufficient medical therapy in our clinic for over 3 months, showed no interval change (Fig. 1a).

**Fig. 2:**
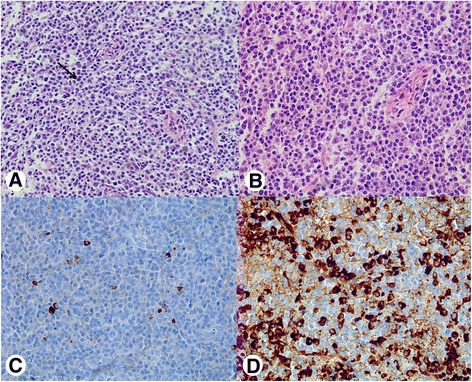
**a**, **b** On microscopic examination, a large amount of lymphoplasmacytic proliferation and infitration (arrow) was observed (**a**, ×200; **b**, ×400). **c**, **d** On immunohistologic examination, tumor cells were negative for kappa stain (**c**, ×400) but positive for lambda stain (**d**, ×400)

An endoscopic biopsy was performed to produce multiple large and deep specimens to differentiate other diseases. Pathologic findings showed dense plasmacytic proliferation of small mature lymphocytic cells; these proliferative cells stained immunohistochemically positive for lambda light chains and negative for kappa chains, suggesting plasmacytoma (Fig. 2). Therefore, we evaluated the patient for MM with a bone marrow biopsy and considered serum monoclonal gammopathy. However, she had no evidence of bone marrow involvement with plasmacytoma or any monoclonal gammopathy through electrophoresis and immunofixation.

We concluded that she had primary gastric plasmacytoma; we recommended local radiotherapy or surgical resection. However, she and her attendant refused further treatment due to old age and limited finances.

She came to our emergency room 3 months later because of hematemesis due to a large gastric ulcer, despite PPI treatment for over 3 months at a local clinic (Fig. 1b). We performed endoscopic hemocoagulation with high-dose PPI and tranexamic acid. We again recommended local radiation or surgical resection, but she still declined extensive treatment. Therefore, we recommended high-dose dexamethasone (40 mg daily for 4 days every 3 weeks) to be administered intravenously to control the plasmacytoma, and she accepted.

After 2 weeks of high-dose dexamethasone, an endoscopy revealed marked healing of her ulcer (Fig. 1c). After a total of four cycles of high-dose dexamethasone, the ulcer was not apparent on endoscopy (Fig. 1d). The patient underwent a total of six cycles of high-dose dexamethasone. One year later, no ulcer was observed on endoscopy.

## Discussion

EMP is a mass of neoplastic monoclonal plasma cells that presents as a solitary lesion in a soft tissue other than the bone. It is uncommon and accounts for approximately 3 % of all plasma cell neoplasms [2].

On endoscopy, gastric plasmacytoma usually appears either as an ulcerated mass or as a discrete ulcer with thickened gastric folds. Thus, it must be differentiated from gastric ulcer, gastric adenocarcinoma, or lymphoma, in particular from mucosa-associated lymphoid tissue (MALT) lymphoma [3, 4]. This case initially revealed extensive mucosal erythema with an ulcer crater and then changed into a large bleeding ulcer.

On pathological examination, a gastric plasmacytoma shows the typical morphologic finding of plasma cell proliferation with immunohistochemistry demonstration of monoclonal light (kappa or lambda) or heavy chains (immunoglobulin G, A, or M). The final diagnosis of primary gastric plasmacytoma is made by histologic confirmation of a single lesion in the stomach without bone lesions, no evidence of plasma malignancy in the bone marrow, and no monoclonal gammopathy [5, 6].

Usually, most EMPs present at an upper respiratory site and are hard to resect completely due to invasiveness; thus, surgery is not recommended for initial treatment. EMP is highly sensitive to radiotherapy; thus, local radiotherapy is the first choice for EMP due to its significant curative potential and lower relapse rate [1].

Currently, there are no general guidelines for the treatment of primary gastric plasmacytoma; however, local radiotherapy may be superior to surgery in plasmacytomas of the stomach due to organ preservation [7, 8].

If primary gastric plasmacytoma only involves the submucosa of the stomach, endoscopic submucosal resection can be another treatment option. If the result of a test for *H. pylori* is positive, then eradication may be an alternative treatment [9, 10]. Recently, bortezomib, which acts as an inhibitor of the ubiquitin–proteasome pathway, alone or in combination with dexamethasone proved effective in the management of extramedullary relapse of MM, and might be an alternative treatment [11].

EMP usually behaves like an indolent disease because of its tendency to localize and it shows a low rate of progression to MM. The prognosis of gastric plasmacytoma is not well understood. However, EMP has a favorable prognosis with a 70 % disease-free survival at 10 years; the 10-year rate of progression of EMP to MM ranges from 11 to 30 % [12, 13]. In this case, our patient and her attendant refused local radiotherapy and surgery due to old age and limited finances; however, she was refractory to ulcer medication and had a life-threatening condition. Therefore, we only used high-dose dexamethasone and the large refractory ulcer showed dramatic regression.

## Conclusions

This is the report of a case of an intractable gastric ulcer, despite repeated endoscopic biopsies and sufficient gastric ulcer therapy, which was finally confirmed as a primary gastric plasmacytoma through deep multiple endoscopic biopsies. The patient only accepted high-dose dexamethasone despite the recommendation of standard local radiotherapy or surgical resection. The intractable and life-threatening ulcer completely regressed and remission was maintained for over 1 year. This case was rare and to the best of our knowledge it is the first report of successful treatment using only high-dose dexamethasone.

## Consent

Written informed consent was obtained from the patient for publication of this case report and any accompanying images. A copy of the written consent is available for review by the Editor-in-Chief of this journal.

## Abbreviations

EMP: extramedullary plasmacytoma
*H. pylori*: *Helicobacter pylori*
MALT: mucosa-associated lymphoid tissue
MM: multiple myeloma
PPI: proton pump inhibitor
SPB: plasmacytoma of the bone
